# Prone versus supine free-breathing for right-sided whole breast radiotherapy

**DOI:** 10.1038/s41598-021-04385-3

**Published:** 2022-01-11

**Authors:** Odile Fargier-Bochaton, Xinzhuo Wang, Giovanna Dipasquale, Mohamed Laouiti, Melpomeni Kountouri, Olena Gorobets, Nam P. Nguyen, Raymond Miralbell, Vincent Vinh-Hung

**Affiliations:** 1 Radiation Oncology Department, Geneva University Hospitals, Geneva, Switzerland; 2 Radiation Oncology, Tianjin Union Medical Center, Tianjin, 300121 China; 3 Service de Radio-Oncologie, Hôpital Riviera-Chablais, Rennaz, Switzerland; 4 CHU de Martinique, Fort-de-France, Martinique France; 5 Radiation Oncology, Howard University, Washington, DC USA; 6 Université de Genève, Geneva, Switzerland; 7 Centro de Protonterapia Quirónsalud, Pozuelo de Alarcón, 28223 Madrid, Spain; 8 Servei de Radiooncologia, Institut Oncològic Teknon, Quironsalud, Vilana 12, 08022 Barcelona, Spain

**Keywords:** Breast cancer, Radiotherapy, Breast cancer, Applied mathematics

## Abstract

Prone setup has been advocated to improve organ sparing in whole breast radiotherapy without impairing breast coverage. We evaluate the dosimetric advantage of prone setup for the right breast and look for predictors of the gain. Right breast cancer patients treated in 2010–2013 who had a dual supine and prone planning were retrospectively identified. A penalty score was computed from the mean absolute dose deviation to heart, lungs, breasts, and tumor bed for each patient's supine and prone plan. Dosimetric advantage of prone was assessed by the reduction of penalty score from supine to prone. The effect of patients' characteristics on the reduction of penalty was analyzed using robust linear regression. A total of 146 patients with right breast dual plans were identified. Prone compared to supine reduced the penalty score in 119 patients (81.5%). Lung doses were reduced by 70.8%, from 4.8 Gy supine to 1.4 Gy prone. Among patient's characteristics, the only significant predictors were the breast volumes, but no cutoff could identify when prone would be less advantageous than supine. Prone was associated with a dosimetric advantage in most patients. It sets a benchmark of achievable lung dose reduction.

Trial registration: ClinicalTrials.gov NCT02237469, HUGProne, September 11, 2014, retrospectively registered.

## Introduction

As in many disease conditions, cancer therapy faces the challenge of improving disease control without increasing toxicity. Radiotherapy has repeatedly been shown to reduce the risk of recurrence in breast cancer. The survival benefit has been modest. Most notably in older clinical trials, the advantage of local control was offset by the risk of heart and lung toxicity^1^. Prone breast radiotherapy is among the techniques that have been developed to reduce these risks^2,3^. However, despite several publications demonstrating the feasibility and the dosimetric advantage for heart and lung^4–7^, prone has been largely ignored in radiotherapy practice. Very few centres have a large volume of prone patients to coach on the technique. There is need that they share their experience, if only for others to appraise when prone can be applied.

In 2010–2013 dual prone and supine dosimetry at the same time for whole breast radiotherapy was implemented as routine practice at the Geneva University Hospitals. Treatment was delivered according to patient's preference and according to the best dosimetry. In a comparison with supine setup in deep inspiration breath hold for left breast cancer, chart review showed a dosimetric advantage associated with prone setup in 62.1% of 116 patients^8^. The comparison used a penalty score that weighted the mean absolute dose deviations (MADD)^9^ to the heart, the lungs, the breasts, and the tumor bed (MADD and penalty weights are detailed in “Methods”). Breast plasticity or pendulousness measured by the change of breast depth between prone and supine, as a ratio or as an absolute difference, and breast volume were significant predictors of an advantage prone. The study also identified how changes of penalty weights affected the dosimetric assessments.

The question naturally arises whether it would be worth the same scrutiny effort to analyze and to report on the dosimetry of the right breast. While attention has been given to radiotherapy of the left breast as in the above study, prone treatment of the right breast has been out of focus. As of January 2021, out of 56 published series of prone and supine dual planning, 7 did not mention the laterality, 31 mixed left and right, 18 were exclusively left, none was devoted to highlight treatment of the right breast. It appears opportune to fill in that gap. The present study aims are:

1. Evaluate the dosimetric advantage of a change of treatment position from supine to prone in right whole-breast radiotherapy, advantage defined as the lowest radiation dose to non-target organs (heart, lungs, contralateral breast) while delivering the prescribed dose to tumor bed and ipsilateral breast, quantified using the penalty score previously applied in left breast analyses^8^.
2. Look for predictors of the dosimetric advantage.
3. Assess the validity of left breast cutoff-predictors of dosimetric advantage when applied to right breast treatment.
4. Examine the effect of penalty weights on the scoring of the radiotherapy treatment plans.

Addressing these aims might add to the evidence of whether or not prone should be considered in breast radiotherapy and might help to identify issues in need of further research.

## Results

### Patients' characteristics

From an original list of 299 dual prone-supine breast CT-simulations, 296 were evaluable dual CT-planning, of which 151 right breast treatment plans were available for analyses. Two were repeat CT that were excluded. Five patients had bilateral breast cancer; left and right were planned separately; of these five bilateral cases, three had supine CT in deep inspiration breath-hold and were excluded, the two with supine CT in free breathing were included. Hence, the total study population was composed of 146 patients, including a case with breast augmentation implant.

Most patients presented with stage 0 or I (Table 1). Lymph nodes were involved in 18.3% of the patients, but nodal irradiation was not retained because of low-risk lymph node ratio or concern for shoulder-arm morbidity in the higher risk. Age presented a normal distribution comparable to Swiss registry data. BMI overweight and obesity were frequent and represented 55.2% of the non-missing records. Patients preferred supine setup in 53.4% of the cases, while 46.6% preferred prone or were indifferent.

**Table 1:** Patient's characteristics.

Characteristic	Subgroup	N (Total = 146)	%
Age (years)	< 50	41	28.1
	50, < 65	56	38.4
	≥ 65	49	33.6
Pathological stage	0	25	17.1
	I	76	52.1
	II	43	29.5
	III	2	1.4
pT	Tis	24	16.4
	T0	2	1.4
	T1	88	60.3
	T2	28	19.2
	T3	4	2.7
Lymph node ratio	Missing	26	
	0	98	81.7
	> 0, 0.20	17	14.2
	> 0.20, 0.65	4	3.3
	> 0.65	1	0.8
Body mass index (kg/m^2^)	Missing	21	
	< 25	56	44.8
	25, < 30	42	33.6
	30, < 35	21	16.8
	≥ 35	6	4.8
Weight (kg)	Missing	10	
	< 60	35	25.7
	60, < 70	46	33.8
	70, < 80	25	18.4
	80, < 90	18	13.2
	≥ 90	12	8.8
Tumor location	Lower inner (LI)	5	3.4
	Central (Cen)	13	8.9
	Upper inner (UI)	18	12.3
	Upper outer (UO)	59	40.4
	Lower outer (LO)	12	8.2
	Other (Oth: overlapping or unspecified)	39	26.7
Heart volume (mL)	< 400	16	11.0
	400, < 500	53	36.3
	500, < 600	53	36.3
	≥ 600	24	16.4
Right breast volume (mL)	< 200	6	4.1
	200, < 400	42	28.8
	400, < 600	43	29.5
	600, < 800	20	13.7
	800, < 1000	19	13.0
	≥ 1000	16	11.0
Couch type	Bionix	113	77.4
	Varian	33	22.6
Patient's preference	Missing	43	
	Supine	55	53.4
	No preference	25	24.3
	Prone	23	22.3
Treatment applied	Supine	63	43.2
	Prone	83	56.8

### Dose-volume histograms and mean absolute dose deviations

**Figure 1:**
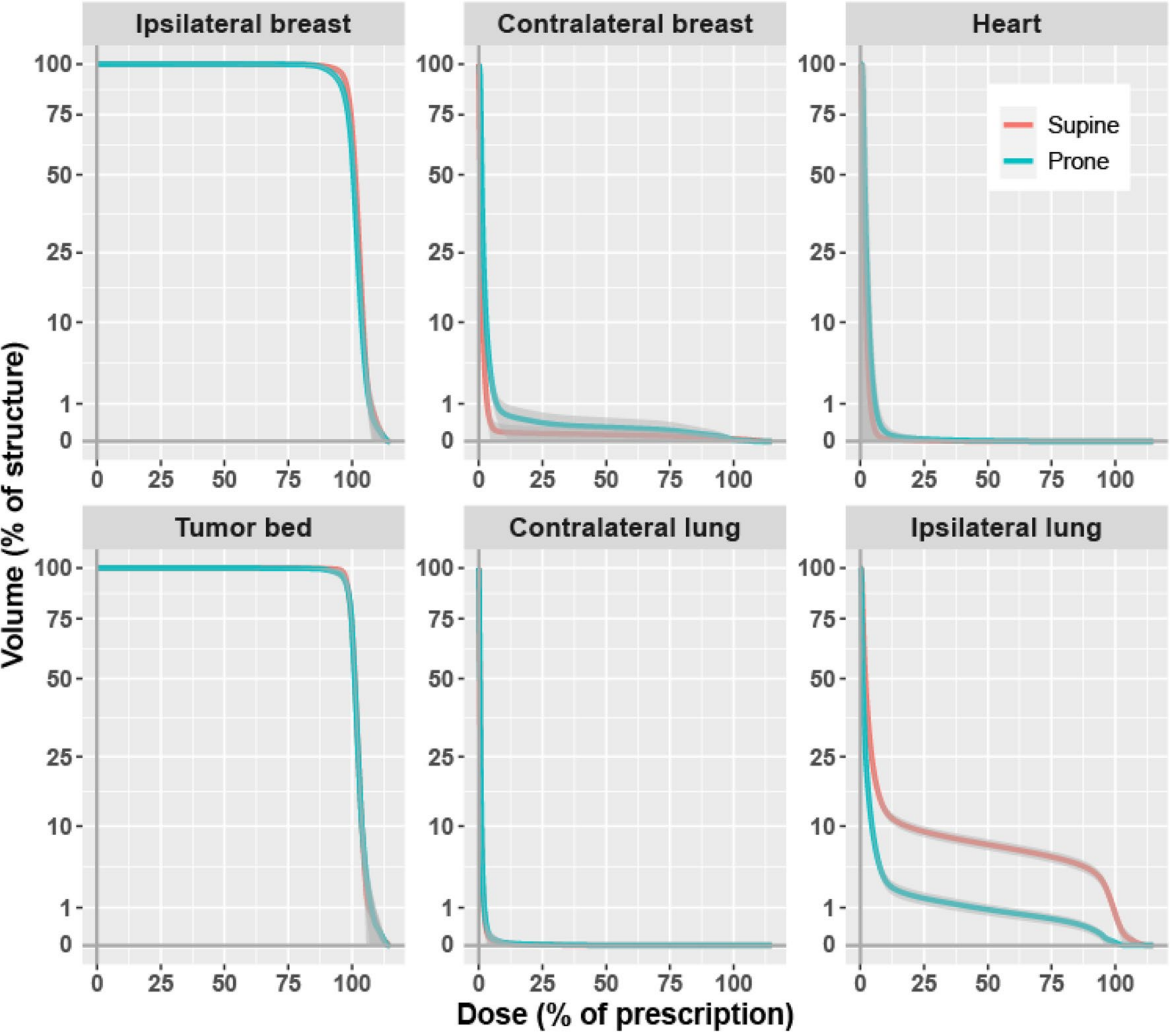
Averaged cumulative dose-volume histograms by structure and setup. Volume, square root scale. Dark grey, 99% confidence band.

**Figure 2:**
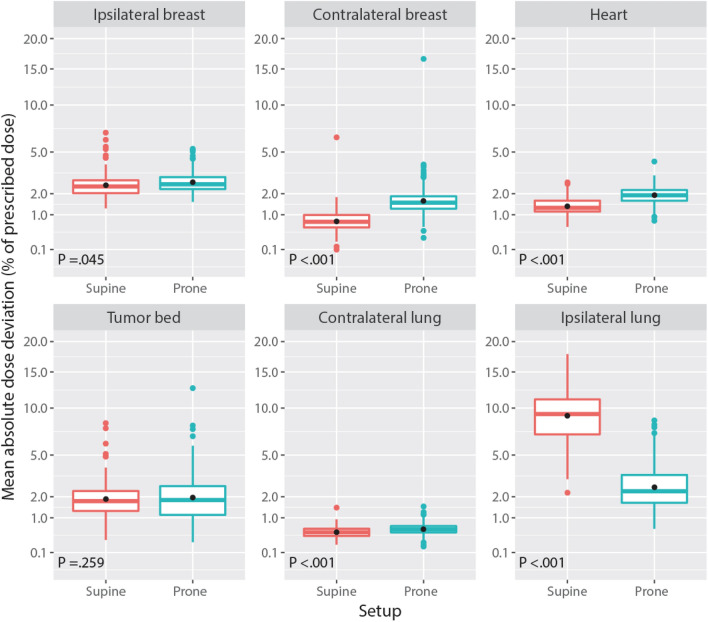
Mean absolute dose deviation (MADD) by structure and setup. MADD axis: square root scale. Box: lower quartile, median, upper quartile. Black dot: average of the MADDs. Color dots: outliers.

Figure 1 shows the pooled cumulative dose-volume histograms for the 6 structures of interest. Prone was associated with a small increase of the doses to the contralateral breast, and a large decrease of the doses to the ipsilateral lung. Figure 2 summarizes the corresponding MADDs. The change from supine to prone was associated with a minimal increase of dose-deviation to the ipsilateral breast PTV, Δ =  + 0.2 percent of dose prescription (PoDP), P = 0.045, the contralateral breast, Δ =  + 0.9 PoDP, P < 0.001, the contralateral lung, Δ =  + 0.1 PoDP, P < 0.001, and the heart, Δ =  + 0.6 PoDP, P < 0.001. The difference, however, was not significant for the tumor bed, Δ =  + 0.2 PoDP, P = 0.259. For the ipsilateral lung, the change from supine to prone was associated with a large decrease of the MADD, from an average of 9.6 PoDP supine to 2.8 PoDP prone, Δ =  − 6.8 PoDP, P < 0.001 (Table 2).

**Table 2:** Mean absolute dose deviation (MADD) by structure and setup.

	MADD supine, PoDP	MADD prone, PoDP	Δ MADD prone–supine, PoDP	P-value
**Heart**				
Mean (SD)	1.4 (0.4)	2.0 (0.5)	0.6 (0.5)	< 0.001
Median (Range)	1.3 (0.6, 2.7)	1.9 (0.8, 4.2)	0.5 (− 0.8, 2.6)	
**Contralateral lung**				
Mean (SD)	0.5 (0.2)	0.6 (0.2)	0.1 (0.2)	< 0.001
Median (Range)	0.5 (0.2, 1.4)	0.6 (0.2, 1.5)	0.1 (− 0.9, 1.2)	
**Ipsilateral lung**				
Mean (SD)	9.6 (3.7)	2.8 (1.6)	− 6.8 (3.6)	< 0.001
Median (Range)	9.3 (2.2, 24.7)	2.3 (0.6, 8.5)	− 6.3 (− 22.2, 0.6)	
**Contralateral breast**				
Mean (SD)	0.8 (0.6)	1.7 (1.4)	0.9 (1.4)	< 0.001
Median (Range)	0.7 (0.1, 6.4)	1.5 (0.3, 16.6)	0.8 (− 4.5, 15.5)	
**Ipsilateral breast**				
Mean (SD)	2.6 (0.9)	2.7 (0.7)	0.2 (0.9)	0.045
Median (Range)	2.4 (1.3, 6.9)	2.5 (1.6, 5.3)	0.2 (− 3.7, 3.0)	
**Tumor bed**				
Mean (SD)	2.0 (1.2)	2.2 (1.7)	0.2 (2.0)	0.259
Median (Range)	1.8 (0.3, 8.2)	1.8 (0.3, 12.6)	0.1 (− 6.1, 10.5)	

*PoDP* percent of dose prescribed.

### Penalty scores

The type-1 penalty scores averaged 2.52 PoDP (range 1.14, 5.52) with supine setup, *versus* 1.93 PoDP (range 0.84, 3.68) with prone setup, 2-sided P < 0.001. The reduction of penalty with prone setup was observed in 119 of 146 patients, 81.5% (binomial 95% confidence interval (CI): 74.2, 87.4). Among these 119 patients, the average expected reduction of penalty from supine to prone was 0.80 PoDP (range: 0.002, 2.99). Among the other 27 patients whose penalty increased with the change from supine to prone, the average expected increase of penalty was 0.33 PoDP (range: 0.031, 1.16).

**Figure 3:**
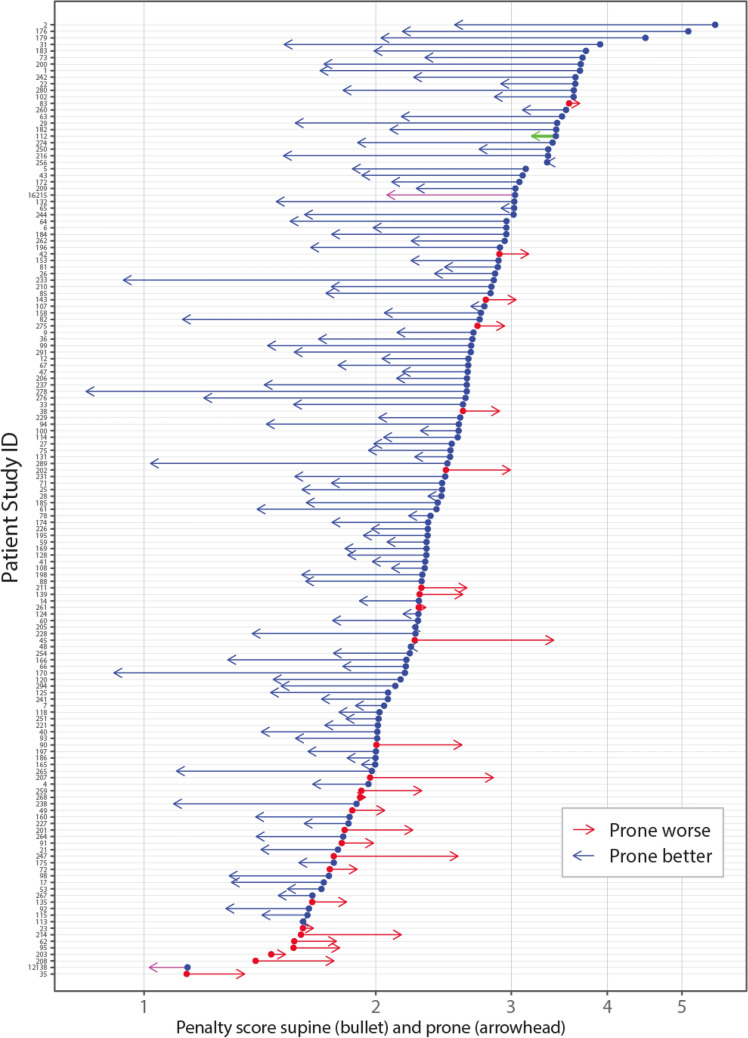
Change of individual patients' penalty score from supine to prone. Bullet, penalty supine. Arrowhead, penalty prone. Arrow right, prone increases penalty; arrow left, prone decreases penalty. Green, breast implant; purple, bilateral cases.

Figure 3 displays the individual patients' penalty scores and the change from supine to prone. There is a clear dependence of the benefit prone with the penalty supine. A high penalty in supine position was predictive of a better penalty in prone position. Linear regression suggested a cutoff at 1.7 PoDP (Table 3). Fourteen patients had a penalty supine less than 1.7 PoDP. Prone reduced the penalty in 6 (42.9%) of these 14, as compared with a reduction in 113 (85.7%) of 132 patients who had a penalty supine of 1.7 PoDP or more, P < 0.001 (Table not shown).

**Table 3:** Robust linear regression predictors of a supine advantage.

Characteristic	Intercept	Coefficient	Standard error	Range supine better	Unit
**Pre-dosimetry**					
Plasticity (pendulousness)					
Ratio breast depth prone/supine	− 0.056	− 0.272	0.222	< − 0.2, NAP	Ratio
Breast depth difference prone − supine	− 0.203	− 0.073	0.030	< − 2.8, NAP	cm
Breast depth prone	− 0.200	− 0.031	0.015	< − 6.5, NAP	cm
Breast/body					
Breast volume/body weight ratio	− 0.358	− 0.026	0.016	< − 13.7, NAP	mL/kg
Tumor location					
Lower inner quadrant vs. else	− 0.537	− 0.164	0.313	< − 3.3, NAP	Binary
Breast size					
Right breast volume supine	− 0.362	− 0.320	0.183	< − 1.1, NAP	L
Left breast volume supine	− 0.406	− 0.191	0.157	< − 2.1, NAP	L
Breast depth supine	− 0.316	− 0.034	0.025	< − 9.3, NAP	cm
Inspiration breath-hold capability					
Right Lung volume supine	− 0.776	0.154	0.182	> 5.0, NEN	L
Total Lung volume supine	− 0.778	0.086	0.098	> 9.1, NEN	L
Left Lung volume supine	− 0.745	0.166	0.194	> 4.5, NEN	L
Age	− 0.651	0.002	0.005	> 338, NEN	Years
Left anterior descending coronary-chest wall distance	− 0.565	0.021	0.087	> 26.9, NEN	cm
Body size					
Weight	− 0.274	− 0.004	0.004	< − 63.8, NAP	kg
Body Mass Index	− 0.185	− 0.015	0.013	< − 12.4, NAP	kg/m^2^
Heart volume supine	− 0.999	0.878	0.598	> 1.1, NEN	L
Height	− 3.028	1.514	0.943	> 2.0	m
Other					
Couch type Varian vs. Bionix	− 0.524	− 0.069	0.142	< − 7.6, NAP	Binary
Preference prone vs. else	− 0.586	0.155	0.160	> 3.8, NAP	Binary
**Post-dosimetry**					
Penalty prone	− 1.619	0.563	0.096	> 2.9	PoDP
Penalty supine	1.290	− 0.763	0.054	< 1.7	PoDP

Range Supine Better computed from the intercept and coefficient to predict when the penalty score changes in favor of supine. *NAP* not attainable physically, e.g. length < 0. *NEN* not expected normally, e.g. age > 338 years.

*PoDP* percent of dose prescription.

**Figure 4:**
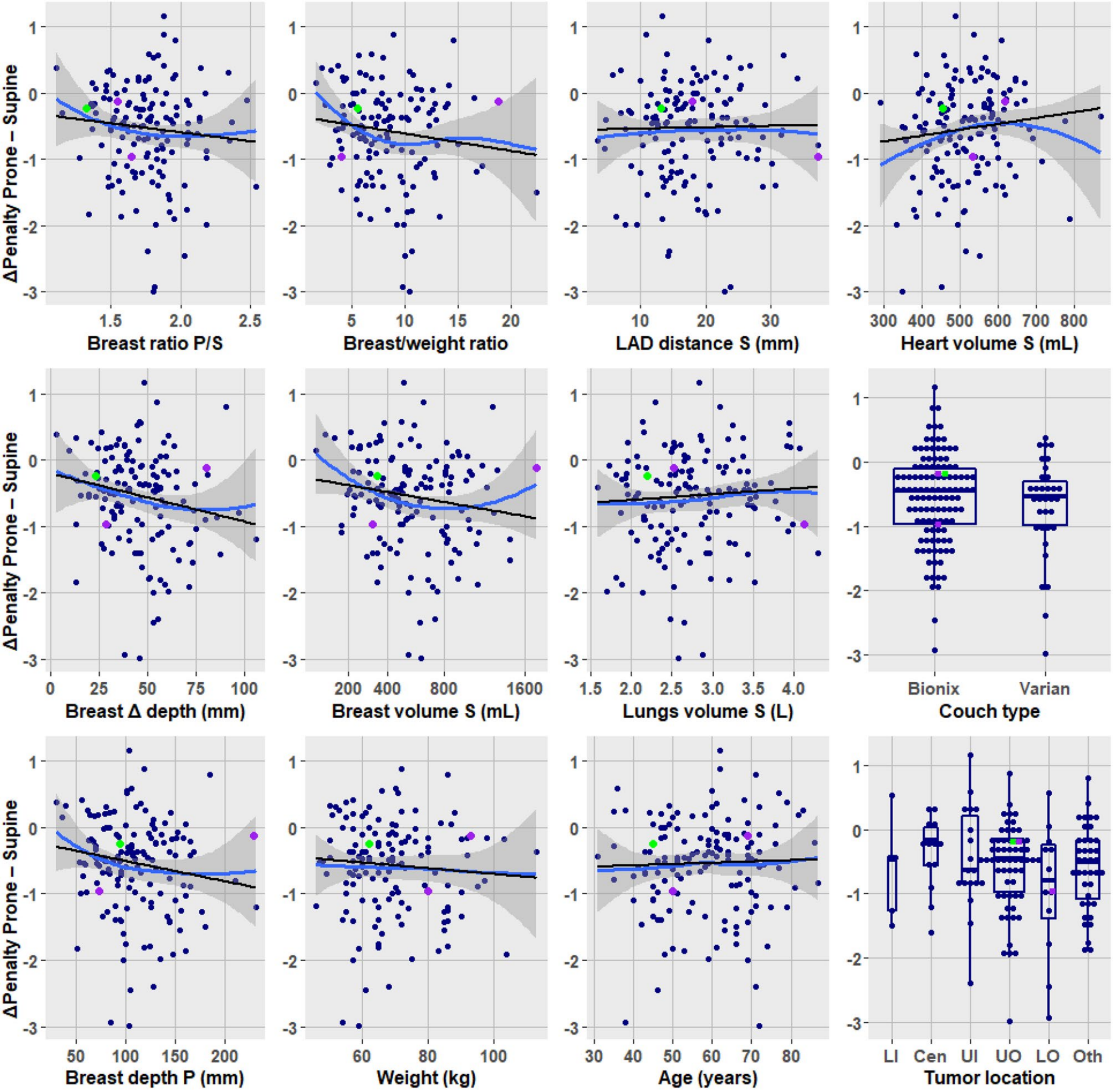
The ΔPenalty prone–supine as a function of characteristics. Negative ΔPenalty indicates a dosimetric advantage with prone (reduction of penalty). Black line, robust linear regression. Blue curve, local polynomial smooth fit (display shows no major departure of linearity). Grey band, 95% confidence. Green, breast implant; purple, bilateral cases. Tumor location: abbreviations in Table 1.

Inversely, a high post-dosimetry penalty score in prone position predicted a dosimetric gain when changing to a supine position (Table 3). But no pre-dosimetry predictor could be formally identified among any of the patients' characteristics. Within the range of physically observable measurements, prone was better across all values of the characteristics. Figure 4 displays the overall preponderance of reduced penalty with prone (negative ΔPenalty).

### Predictors of Δ penalty change

Table 4 examined the applicability of the cutoffs previously found significant for the left breast^8^. Indices of pendulousness were not significant, whereas measures related to breast volume were significant: ratio of breast volume over body weight, breast volume proper, and depth of the right breast supine. Note that the significance and the odds ratios in Table 4 relate to the steepness of the relationship between the characteristics and the Δ penalty prone shown in Fig. 4. These validate the cutoffs. But the prone advantage remains preponderant over all the pre-dosimetry subgroups defined by the cutoffs. Thus, in Table 4, the column 'Prone better' shows in all rows a prevalence higher than that of the column 'Supine better'. This corresponds well with the plot panels of Fig. 4 which show trends (i.e. there is a relationship between the predictor of interest and the Δ penalty prone), but nevertheless all fits remained below 0 (i.e. the trends were not enough to negate the advantage prone).

**Table 4:** Dosimetric gain by cutoff derived from left dataset applied to the right breast.

Characteristic	Supine better N = 27	Prone better N = 119	Odds ratio prone better	P value
**Pre-dosimetry**				
Ratio breast depth prone/supine				0.552
≤ 1.6	9 (22.0%)	32 (78.0%)	0.76	
> 1.6	18 (17.6%)	84 (82.4%)	1.31	
Breast depth difference prone − supine				0.220
≤ 31 mm	8 (26.7%)	22 (73.3%)	0.56	
> 31 mm	19 (16.8%)	94 (83.2%)	1.80	
Breast depth prone				0.180
≤ 77 mm	8 (27.6%)	21 (72.4%)	0.53	
> 77 mm	19 (16.7%)	95 (83.3%)	1.90	
Breast volume/Body weight Ratio				0.012
≤ 4.9 mL/kg	8 (36.4%)	14 (63.6%)	0.29	
> 4.9 mL/kg	16 (14.0%)	98 (86.0%)	3.50	
Lower inner quadrant (LIQ)				0.930
LIQ	1 (20.0%)	4 (80.0%)	0.90	
Not LIQ	26 (18.4%)	115 (81.6%)	1.11	
Right breast volume supine				0.004
≤ 282 mL	8 (42.1%)	11 (57.9%)	0.24	
> 282 mL	19 (15.0%)	108 (85.0%)	4.13	
Left breast volume supine				0.164
≤ 347 mL	5 (31.2%)	11 (68.8%)	0.45	
> 347 mL	22 (16.9%)	108 (83.1%)	2.23	
Breast depth supine				0.035
≤ 38 mm	5 (41.7%)	7 (58.3%)	0.28	
> 38 mm	22 (16.8%)	109 (83.2%)	3.54	
**Post-dosimetry**				
Penalty score prone				< 0.001
≥ 2.4 PoDP	12 (54.5%)	10 (45.5%)	0.11	
< 2.4 PoDP	15 (12.1%)	109 (87.9%)	8.72	
Penalty score supine				< 0.001
≤ 2.1 PoDP	17 (38.6%)	27 (61.4%)	0.17	
> 2.1 PoDP	10 (9.8%)	92 (90.2%)	5.79	

*PoDP* percent of dose prescription. Breast not specified is the right (ipsilateral) breast.

### Effect of modifying penalty weights

**Figure 5:**
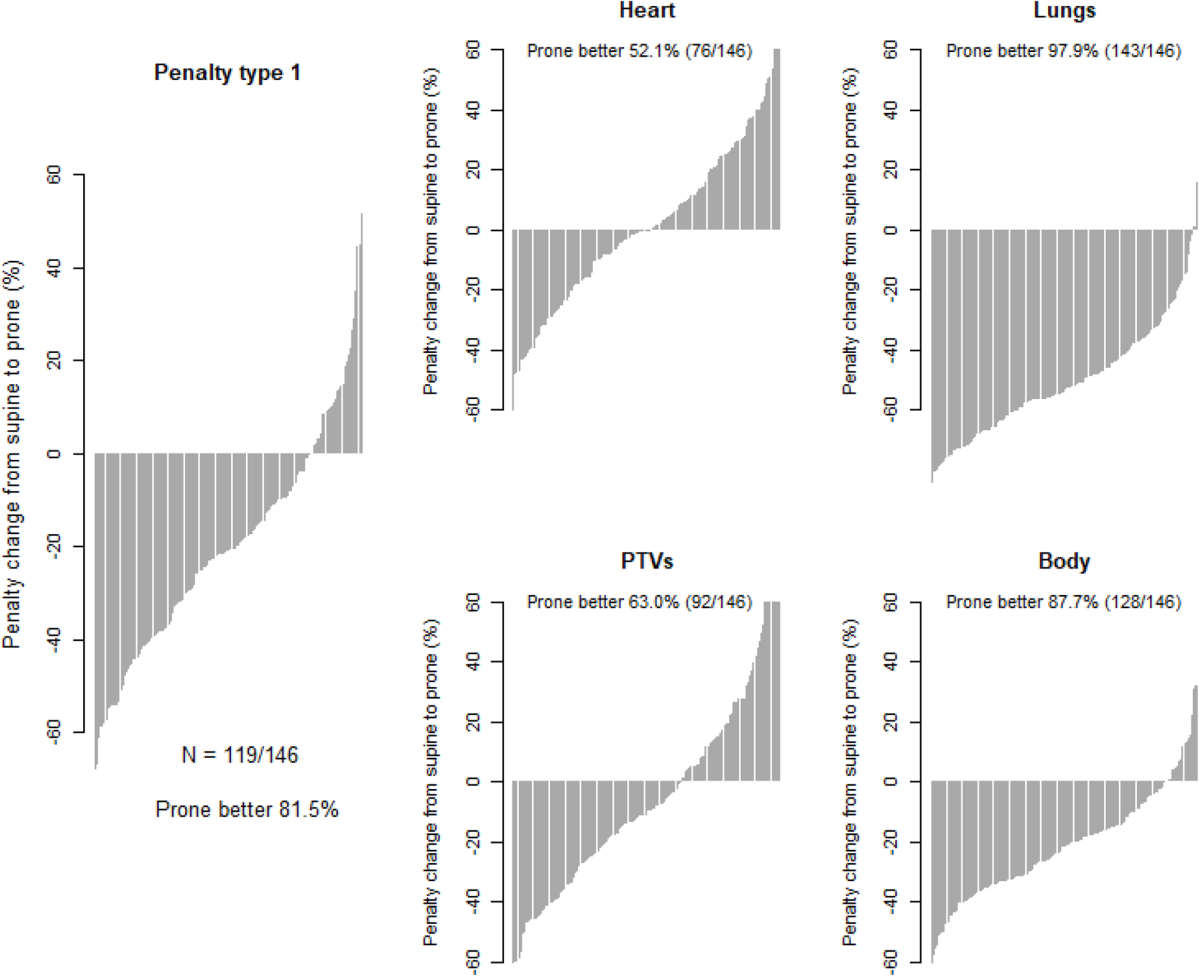
Waterfall plot of percent penalty change from supine to prone.

The waterfall plots of Fig. 5 evaluated how different penalty weights (Table 5) affected the distribution of the penalty reductions or increases when switching from supine to prone. Prone was associated with a reduction of penalty in 52.1% (95% CI: 43.6, 60.4) of the patients when the heart was assigned two-third of all the priority weights, such as would be required in case of cardiac morbidity^10,11^. With priorities assigned to PTVs^12,13^, prone advantage was observed in 63.0% (95% CI: 54.6, 70.8) patients. With priorities assigned to the lungs^14,15^, prone was better in 97.9% (95% CI: 94.1, 99.6) patients. With priority assigned to the body on the whole as might occur when concern is the risk of radiation induced tumors^16–18^, 87.7% (95% CI: 81.2, 92.5) of the patients derived a benefit with prone.

**Table 5:** Alternative sets of penalty weights.

Priority type	Weights						
	Heart	Contralateral Lung	Ipsilateral Lung	Tumor bed	Ipsilateral Breast	Contralateral Breast	Body
Type 1	**0.40**	**0.16**	**0.14**	0.11	0.09	0.10	0
Heart	**0.65**	0.08	0.07	0.10	0.05	0.05	0
Lungs	0.20	**0.30**	**0.35**	0.07	0.04	0.04	0
PTVs	0.15	0.08	0.07	**0.35**	**0.30**	0.05	0
Body	0.10	**0.15**	**0.15**	0.08	0.07	**0.15**	**0.30**

Transposed from Table 3 in: Wang X et al.^8^. Reproduced without change according to a Creative Commons Attribution 4.0 International License.

Each row indicates a penalty type. The weights assigned to the structures related to a given priority type are chosen to sum to ≥ 0.65, highlighted in bold. PTVs, planning target volumes.

## Discussion

The vast majority of patients worldwide receive adjuvant radiotherapy for breast cancer in supine position although it is known that the prone position offers anatomical advantages which can be exploited to decrease dose to organs-at-risk. Evidence-based techniques to reduce risk of radiation-related toxicity include intensity modulation (IMRT), hypofractionation, deep-inspiration breath hold and prone position. Widespread implementation of 3 of these techniques exists but the use of prone position is scarce. In a Spanish survey, only 3 of 40 centers rarely used prone position^19^. In German-speaking countries, 1 (1.47%) of 68 (imputed) surveyed radiotherapy department offering heart-sparing used prone^20^. Among 3894 patients receiving unilateral whole breast radiotherapy in 2014–2018 at the Sunnybrook Health Sciences Centre, 80 (2.1%) were treated prone^21^. From the Michigan Radiation Oncology Quality Consortium, 200 (4.3%) of 4688 breast cancer patients were treated prone^22^. A dosimetric database review from Brisbane, Australia, identified only 13 (1.8%) patients actually treated with prone breast radiotherapy for 708 supine treatments^23^. The evidence of low uptake has important implications. The concept of prone radiotherapy is simple—in prone setup, the breast hangs down from the chest wall through an aperture on the support couch, facilitating avoidance of intrathoracic organs with radiation tangential fields—but the implementation is not. It requires the full radiation team to collaborate on many levels, to address the challenges of implementing prone setup that include, among other: comfort of the patient (53.4% preferred supine, Table 1), stability and set-up precision using the present prone devices, the need for new devices^24^, the longer set-up times. Surface-guided set-up systems reported for supine^25^ are not well suited for prone breast positioning. Prone breast treatment is not part of the curriculum of radiation oncologists, physicist or technologists, which might impact the radiotherapy flow process, from delineation of the index breast when prone to treatment delivery with adequate alignment of the patient^2^. There is no (or negative) financial incentive for change of practice to prone position. With 10 patients per year in a centre, the technique cannot be truly developed. Prone is an untapped resource to improve patient care^26^.

Originally proposed for large breasted women^27^, the restriction of prone to large volumes has been challenged^28–32^. Like the latter authors, we observed a dosimetric benefit in most patients irrespective of breast volume. There is a highly significant relationship between breast volume and prone advantage. However, the magnitude of the advantage is such that the percentage prevalence of prone benefit surpasses the prevalence of supine benefit even with a breast volume as small as 282 mL (Table 4). The ratio of breast volume on body weight and the breast depth correlated with breast volume, they show the same relationship with prone advantage.

In contrast to the left breast study, none of the pendulousness indicators were significant. In left breast radiotherapy, the benefit of prone is affected by how much the breast displaces from the heart, hence is dependent on the plasticity of the breast. In right breast radiotherapy, the distance between the tangential field's edge and the heart is less affected by how the gantry is adapted to the breast, hence the benefit remains independent of pendulousness.

**Figure 6:**
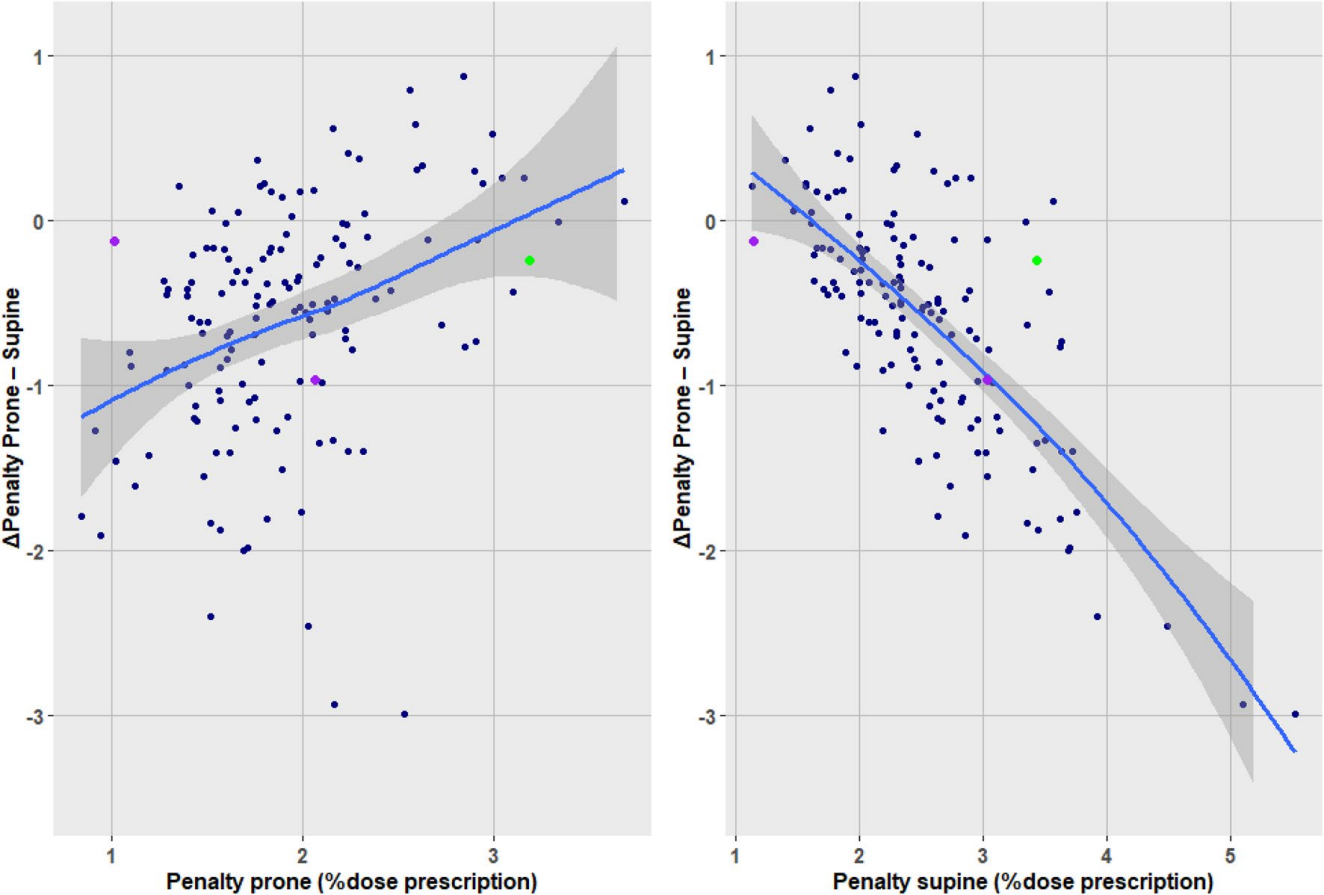
The ΔPenalty as a function of penalty prone or supine. Blue curve, local polynomial fit. Grey band, 95% confidence. Green, breast implant; purple, bilateral cases.

The strongest predictors were the post-dosimetry penalty scores, as shown with the regression results of Table 3 and depicted in Fig. 6. That applies when a single treatment plan has been performed. Let us consider, in accordance with other authors^2,33,34^, a patient undergoing a single CT-simulation in a prone position. A prone plan penalty of ≥ 2.4 PoDP, based on left breast data^8^, or > 2.9 PoDP, based on the present right breast data (Table 3), would yield a prevalence of ≥ 54.5% supine plans better than prone (Table 4), which would justify to re-simulate the patient supine, while a prone plan penalty of < 2.4 PoDP, giving an odds ratio of 8.72 in favor of prone, would not.

The MADDs pertaining to the right breast plans (Fig. 2) mirror those of the left breast previously reported (Fig. 4 in^8^). The difference pertains to heart doses that were higher with the left breast. The previous left side median heart MADD were 3.4 prone and 1.9 supine in deep inspiration breath-hold, as compared with the present right side median heart MADD 1.9 prone and 1.3 supine free-breathing (Table 2). Between right and left, the left side higher heart doses prone and supine, (3.4 and 1.9) > (1.9 and 1.3), is attributable to the heart nearer to the treatment fields. Between prone and supine, the heart ΔMADD difference, larger left (Δ =|3.4–1.9|= 1.5) than right (Δ =|1.9–1.3|= 0.6), is attributable to breath-hold.

The lung doses delivered to the patients in supine position were low. The mean ipsilateral lung dose normalized to a breast prescription of 50 Gy was 4.8 Gy (9.6 PoPD, Table 2). That is nearly half less than the 8.4 Gy reported in the literature for whole breast/chest wall radiotherapy^35^. The low supine lung dose can be ascribed to our choice of no PTV expansion. A dosimetric study comparing 0, 5 and 10 mm PTV expansion from the breast target volume reported a mean dose to combined lungs of 2.0–5.0 Gy, 2.8–5.1 Gy and 3.0–5.2 Gy, respectively^36^, which correspond well with our combined lungs average dose of 2.5 Gy supine. Prone further reduced the ipsilateral lung dose to the equivalent of 1.4 Gy (2.8 PoPD, Table 2). The corresponding proportional reduction of the dose to the ipsilateral lung obtained with prone was 70.8% = [(4.8 Gy–1.4 Gy) ÷ 4.8 Gy] ∙ 100. That is well beyond any achievable reduction with radiotherapy changes of technique in the supine position, typically in the range of 20–30% without nodal irradiation, except perhaps with proton^37,38^.

In the analysis with the Type 1 penalty score, which assigns more than two-third of the penalties to the heart and the combined lungs (Table 5), a dosimetric advantage was found with prone in 81.5% of the patients. That advantage is attributable to the reduction in lung dose, without being offset by increase in heart dose. Note that this implies a supine advantage in 18.5% of the patients, meaning that research to improve techniques supine needs to continue. Modifying penalty weight showed a preponderant advantage of prone in all four types of weights, ranging from 52.1% to 97.9% of the patients (Fig. 5). The prevalence of prone advantage based on our priority Type 1 is lower than reported from the New York University (NYU), where prone was found optimal in all right breast patients (N = 200), none of whom had heart in-field^4^. The next largest series that included right-sided prone treatment without nodal irradiation had N = 47 right breast patients^39^, N = 35^28^, and N = 33^40^. But even though dose differences were provided, the data were confounded with the left breast, the prevalence of a prone advantage could not be extracted from the publications.

**Figure 7:**
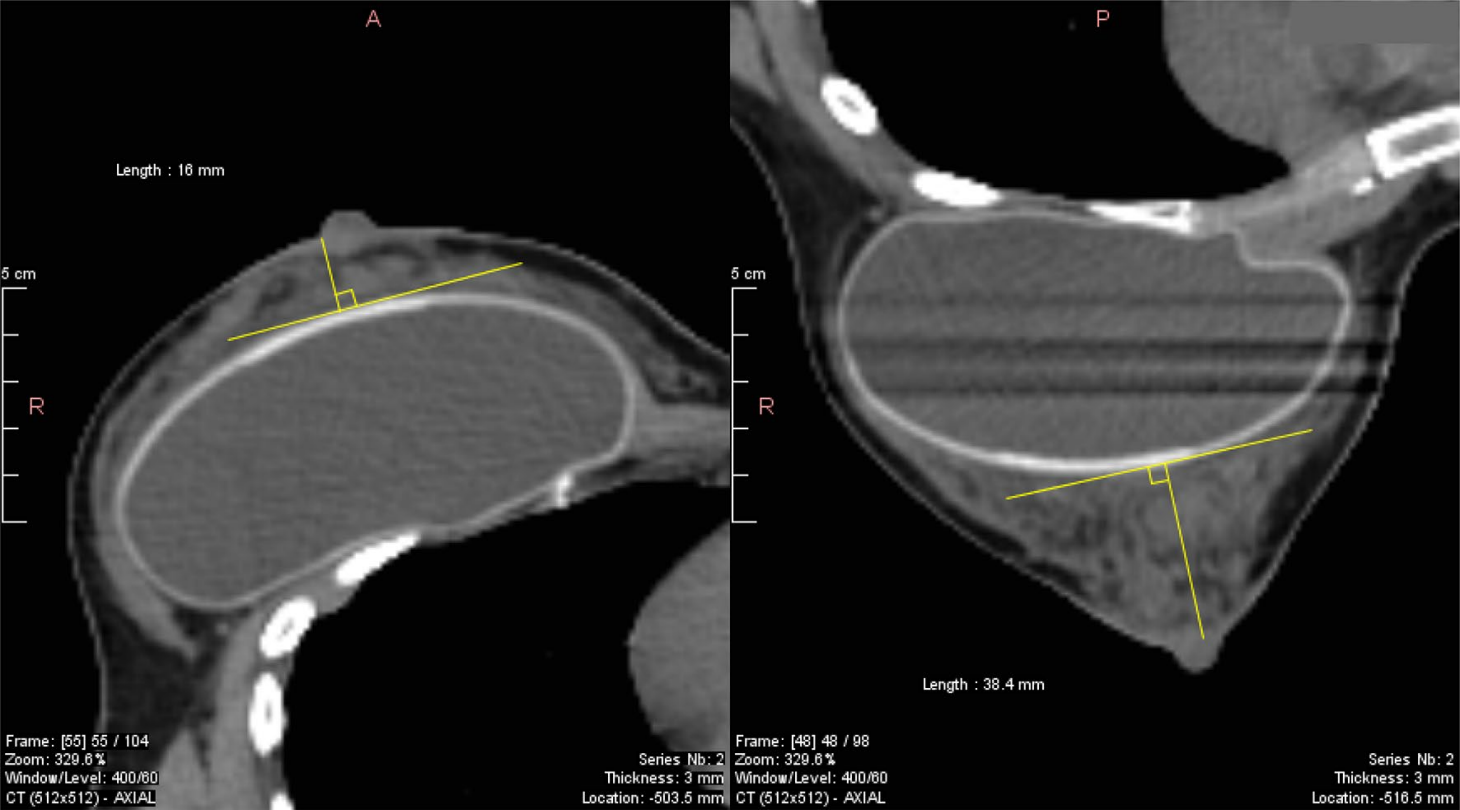
Breast augmentation implant supine and prone. Supine (left) and prone (right) CT of case 112, green color-coded in Figs. 3, 4 and 6. The breast depth measured from the pleura was 71 mm supine vs. 94 mm prone. The depth remeasured from the implant anterior surface is 16 mm supine vs. 38 mm prone, indicating a shift with gravity of > 50%.

The patients' population included one breast augmentation implant and two bilateral cases. Analysis with or without these cases did not affect the overall results. They are identified by color-code in Fig. 3, 4, and 6. One of the bilateral cases had the largest breast volume, which affected the non-linear fit, but not the linear regression. All 3 patients derived a small benefit with prone. These are unusual situations that might warrant consideration (Fig. 7).

A dosimetric study would be incomplete without a display of dose-volume histograms. Modern radiotherapy planning optimization processes deal with small dose differences, of the order of a few percentage points^41^. We strived to attain graphical integrity, visualizing small differences without undue distortion. Textbook approaches include the logarithmic transforms which are naturally intuitive^42^. However, the logarithm is negative-infinite at 0 and considerably amplifies the very small values. We selected the not-so-trivial square-root transform, of which Wilke commented that unlike the linear or the log scale there is no simple addition-subtraction or multiplication-division rule, and it is unclear how to best place axis ticks on a square-root scale^43^. We found the y-axis square-root preserved the patterns of DVHs with the advantage of a moderate zoom on the small values. The tick marks spacing was reasonably solved in Fig. 1 with the choice of 1, 10 then every 25 units. The arithmetic simplicity concern is addressed by noting that √0 = 0, √1 = 1, thus a distance on the graph's paper of 1 cm (or other printing scale by user choice) corresponds exactly to the DVH's volume marks of 0% to 1%. From there on, a volume of 4% is represented by √4 = 2 cm, 9% by 3 cm, 16% by 4 cm, 25% by 5 cm, and so forth, to 100% represented by a tick mark at 10 cm. All points can be visually identified almost without a calculator or a ruler. For example, if one is interested in the ipsilateral lung V20Gy, which corresponds to 40% of a prescribed dose of 50 Gy, a vertical line at the 40% dose crosses the prone blue line at volume 1%, and crosses the supine red line at about 3 times higher than 1 (Fig. 1 ipsilateral lung panel), corresponding to a volume of 9%. That is, the ipsilateral lung V20Gy is 1% with prone, versus 9% with supine. Graphical integrity was further preserved by repeating axis labels on all panels. Likewise, the square-root transform was applied in Fig. 2, allowing a well-balanced visual detection of tiny differences that would have escaped notice, such as the significant slightly increased heart dose.

The study has limitations that are common with the left breast study^8^. It is retrospective, subject to recollection and data analysis biases. It is tempting to exclude unexpected cases that do not fit the majority, such as the implant and the bilateral cases. Breast depth and left anterior descending distance were measured after planning. There was no registration of patients who could not undergo dual simulation or were unable to be positioned prone, estimated to represent 10–15% of the patients eligible for whole breast radiotherapy. All contours were approved by a single oncologist without blind assessment. The actual treatment given to patients depended on 3-dimensional dose distributions which were not reviewed. Unlike another study, we did not integrate the boost^44^. Missing height and weight data could not be controlled. In addition, the liver was not delineated, which could be relevant in the radiotherapy of the right breast. On review of treatment images, a small part of the liver was apparent on supine portal images, but seldom on prone portals, suggesting that the benefit prone could have been more if a liver penalty had been included. A concern is that the analyses did not use more common metrics, such as the lung V20Gy, the V50% for the heart, or the homogeneity index for the breast PTV. These are important for treatment planning optimization. However, unlike the MADD which summarizes the DVH of any organ or target on the same unit as the prescribed dose, the common metrics are on different units, preventing their direct use as penalty score.

Strengths are also those of the left breast study^8^. All treatment plans were done prospectively with the intent to deliver the best possible plan, regardless of position. Treatment plans were retrieved as-is, without nudging to improve on any shortcoming. The dual-planning setup is that of an exact balanced design in which each patient is her own comparator. Contouring differences that could affect different population were equalized. There was no patient exclusion for breast size or other reason. The data provides a realistic insight into what might be expected with prone dosimetry.

We are aware that the data is abundant. The complexity of the interpretation is commensurate with the interrelated study aims. Herein we present a roadmap of the results. Aim 1, dosimetric advantage or not, was addressed in Figs. 1–3 and Table 2. Aim 2, search of predictors, was addressed in Fig. 4, Fig. 6, and Table 3. Aim 3, do left breast cutoffs apply to prone right breast radiotherapy or not, was addressed in Table 4. Aim 4, how would a change of priorities to organs or targets affect the prone dosimetric advantage was addressed in Fig. 5 and Table 5. Figure 7 is an unexpected unplanned observation. It shows as other breast cases that the breast parenchyma over an implant was also affected by gravity. We hope future studies will accrue more data on breast implants.

Is the prone dosimetric advantage—attributable to a reduction of lung dose without excess dose to other organs and without decrease of dose to targets—sufficient to warrant a change of practice? Early radiation pneumonitis is detectable on follow-up CT 1–3 months after breast cancer radiotherapy^45^. In a photon-proton collaborative study of patients at high risk of breast cancer recurrence, radiobiological modeling estimated the thirty-year mortality rates from radiotherapy-related cardiac injury and lung cancer at 1.66% to 4.03%^41^, although these rates did not outweigh the ~ 8% disease-specific survival benefit of radiotherapy. In a study estimating lung cancer and cardiovascular mortality among female breast cancer patients receiving radiotherapy, the risks were shown to increase with lung and heart dose, even in non-smokers without familial or cardiac history^46^. In a randomized clinical trial comparing two radiotherapy techniques, the experimental arm provided a modest reduction of the lung radiation dose, mean 4.7 Gy, vs. 6.6 Gy in the conventional arm, yet enough to show a significantly preserved lung diffusion capacity at 2 years^47^. At 10 years follow-up, the patient's self-reported outcome evaluation of that trial found in the experimental arm a significantly better survival free from deterioration in any of dyspnea, fatigue, physical functioning, or pain measures^48^. There is plenty evidence that increased dose to organs at risk increases the risk of mortality, and there is emerging evidence that decreased dose is associated with improved outcome.

This study sets a benchmark by which a 70.8% reduction of irradiated lung volume is achievable. Prone provided an overall dosimetric advantage in 81.5% of right breast cancer patients. Breast volume related significantly with the magnitude of prone advantage, but a reliable cutoff by which supine would supersede prone could not be identified. Prone should be available to allow optimal individual patient selection.

## Methods

### Study population

Patients were retrospectively selected from the Geneva University Hospitals’ radiotherapy database. They presented with a primary cancer of the right breast completely resected with breast conserving surgery, were referred between September 2010 and August 2013 for adjuvant radiotherapy, and underwent computed tomography (CT) simulation and treatment planning in prone and supine position, both in free breathing. The study is a chart review of these cases. It received Institutional Review Board approval and was registered under ClinicalTrials.gov Identifier NCT02237469. All treatments were performed in accordance with applicable guidelines and regulations. Informed consent for the management was obtained from all participants.

### Patient setup

As described previously^8^, the patient was positioned supine on an inclined breast board with arms extended above head. Supine CT-images with 3 mm slices were acquired without contrast. The scan range covered the entire lungs and breasts, from the top of the lungs to 5 cm caudal to the breasts or to the base of the lungs, whichever was the most caudal. Thereafter the patient was positioned prone using the *Bionix Prone Breast System* in 2010–2012 and the *Varian Pivotal Prone Breast Care* in 2013. The left (contralateral) breast rested on a 5 degrees foam wedge. The right (ipsilateral) breast was inspected to hang unhindered and centered through an opening in the couch support. Prone CT-images were acquired with the same parameters as supine. Posterior and lateral positioning marks were tattooed. A patient self-assessed questionnaire recorded the patient's subjective feelings of pain, fear, anxiety, and discomfort, and position preference at the end of simulation.

### Treatment planning

The breast clinical target volume (CTV) was delineated cranially up to 1 cm below the sternoclavicular joint, caudally to the farthest visible breast contour, medially to the perforating mammary vessels or to the edge of the sternum, laterally to the lateral breast-skin fold, posteriorly to but not beyond the surface of the pectoralis muscle or ribs and intercostal muscles, anteriorly to 5 mm under the skin surface^49^. The tumor bed CTV was delineated using the patient's clinical, radiological and surgical-pathological data. Planning target volume (PTV) equated CTV without expansion. Delineation of the contralateral breast included the skin surface. Delineation of the heart included the pericardium and the basis of the large vessels^50^, but not above the top of the left atrium. Delineation of the lungs and the body's external contour used automatic segmentation. Treatments were planned without boost using Varian Eclipse.

The radiation delivery technique used two static opposed multi-leaf photon tangential beams aligned on the treatment fields' posterior border. The field borders were specified to cover the breast PTV, extending cranially to 2 cm under the humeral head, 1 cm caudal to the breast, 3 cm anterior to the breast, and posteriorly with maximum 2 cm central lung distance. The beam angles and collimator leaves were applied to fully avoid the heart and the contralateral breast, and to avoid the lung as much as possible. The planning was optimized with constraints of 95% of prescribed dose covering 95% of breast PTV and covering 100% of tumor bed PTV, breast PTV V_107%_ < 2 cc, ipsilateral lung V_20 Gy_ < 10%, heart near max D_2%_ < 15 Gy, and heart mean dose < 3 Gy. Dynamic wedges and field-in-field modulation were applied as needed to meet the constraints. Doses to organs and targets were converted to percentages of the dose prescription, denoted PoDP to avoid confusion with percent change.

### Penalty score, mean absolute dose deviation, priority weights

Treatment plans were assessed by a penalty score defined as^8^:

$$Penalty Score=\sum_{i=1}^{K}{w}_{i}\times {M}_{i}$$

where *i* represents a structure, organ or target, from a list of *K* structures, *w*_*i*_ represents the penalty weight assigned to the structure *i*, and *M*_*i*_ represents the treatment plan's MADD of the structure *i*^9^.

The MADD *M*_*i*_ for a given structure *i* is defined as

$${M}_{i}={\int }_{0}^{V0}\frac{\left|D-\right.\left.A\right|}{V0} dV$$

where *D* represents the dose abscissa, *V* the volume ordinate of the set the cumulative dose-volume histogram (DVH) of the structure, *V0* the volume of the structure, and *A* the reference dose for the structure. Simply, from calculus, *M*_*i*_ is the area between the DVH and *A*. Since *dV*/*V0* units cancel out, *M*_*i*_ unit is the same as that of |*D* − *A*|, a difference of doses.

The set of default weights for the penalty score, denoted "penalty type 1"^8^, was 0.40, 0.16, 0.14, 0.11, 0.10, and 0.09, for the *K* = 6 structures of interest, heart, contralateral lung, ipsilateral lung, tumor bed, ipsilateral breast, and contralateral breast, respectively. These values represent ordinal priorities heart > lungs > CTV tumor bed > contralateral breast > CTV ipsilateral breast. They were converted to a pseudo-continuous scale with the constraint that their sum equals 1. The weights' sum constraint allows expression of the penalty score on the same unit as *M*_*i*_. Other penalty weights will be discussed later.

### Design of the analyses

In the comparison of prone versus supine, prone was considered to provide a dosimetric advantage if the penalty score was reduced by changing from supine to prone. The change in penalty score was assessed by the counts of patients who benefitted from prone and by visual summarizing displays. Robust linear regression^51^ and fractional polynomial regression^52^ were used to evaluate potential predictors of the dosimetric gain: patient's age, height, weight, body mass index (BMI), tumor location, planning breast volume, patient's preference, CT measurements of the breast depth supine and prone (distance from the breast surface to the pleura as defined in^8^), and post-dosimetry penalty scores.

All statistical computations used R version 3.6.3^53^. Visual display used the package *ggplot*^54^. Regression analyses used the packages *MASS*^51^ and *mfp*^52^.

### Ethics approval

The study received approval at the Geneva University Hospitals Institutional Review Board.

### Consent to participate

Informed consent was obtained from all individual participants included in the study.

## Data Availability

The study data is available on Mendeley, Reserved https://doi.org/10.17632/rv7pjnfhxx.1. Temporary link to preview: https://data.mendeley.com/datasets/rv7pjnfhxx/draft?a=6928ed0a-351d-4937-9c0d-a0c8bfd68c99.
